# Synthesis and Early Development of Hexadecyloxypropylcidofovir: An Oral Antipoxvirus Nucleoside Phosphonate

**DOI:** 10.3390/v2102213

**Published:** 2010-09-30

**Authors:** Karl Y. Hostetler

**Affiliations:** 1 Department of Medicine, Division of Infectious Disease, University of California, San Diego, 9500 Gilman Drive, La Jolla, CA 92093-0676, USA; E-Mail: khostetler@ucsd.edu; Tel.: +1-858-552-8585; Fax +1-858-534-6133; 2 Veterans Medical Research Foundation, San Diego, CA 92161, USA

**Keywords:** antiviral drugs, Cidofovir, HPMPA, acyclic nucleoside phosphonates, smallpox, vaccinia, biodefense

## Abstract

Hexadecyloxypropyl-cidofovir (HDP-CDV) is a novel ether lipid conjugate of (*S*)-1-(3-hydroxy-2-phosphonoylmethoxypropyl)-cytosine (CDV) which exhibits a remarkable increase in antiviral activity against orthopoxviruses compared with CDV. In contrast to CDV, HDP-CDV is orally active and lacks the nephrotoxicity of CDV itself. Increased oral bioavailability and increased cellular uptake is facilitated by the lipid portion of the molecule which is responsible for the improved activity profile. The lipid portion of HDP-CDV is cleaved in the cell, releasing CDV which is converted to CDV diphosphate, the active metabolite. HDP-CDV is a highly effective agent against a variety of orthopoxvirus infections in animal models of disease including vaccinia, cowpox, rabbitpox and ectromelia. Its activity was recently demonstrated in a case of human disseminated vaccinia infection after it was added to a multiple drug regimen. In addition to the activity against orthopoxviruses, HDP-CDV (CMX001) is active against all double stranded DNA viruses including CMV, HSV-1, HSV-2, EBV, adenovirus, BK virus, orf, JC, and papilloma viruses, and is under clinical evaluation as a treatment for human infections with these agents.

## 1. Introduction

Smallpox was a major cause of mortality worldwide until it was eliminated by the WHO vaccination program in the late 1970s [1]. It was a disease easily transmitted by aerosol which caused 30% mortality and disfiguring skin lesions in survivors. Vaccination for smallpox was discontinued thereafter and the world population has become vulnerable again to the disease because of minimal herd immunity. Several years ago concern arose that smallpox could be used as a bioweapon by hostile groups or terrorists [2,3]. In addition, genetically modified strains of poxviruses were shown to be lethal to animals even though they had been preimmunized by vaccination [4]. Although vaccination is still considered as the primary approach to a potential recurrence of smallpox, many individuals are now immunosupressed because of HIV infections, cancer chemotherapy and organ transplantation. Immunosuppressed persons would not be good candidates for vaccination [5] and alternative therapies are needed. A screening effort was undertaken to find agents which inhibited the replication of poxviruses and could be developed as therapeutics.

## 2. Cidofovir Identified as an Effective Inhibitor of Poxviruses

(*S*)-1-(3-hydroxy-2-phosphono-methoxypropyl)adenine (HPMPA) and (*S*)-1-(3-hydroxy-2- phosphonomethoxypropyl)-cytosine (HPMPC, cidofovir) are acyclic nucleoside phosphonate antivirals first described by Holy and De Clercq [6,7]. Both HPMPA and HPMPC were shown to have antiviral activity in the vaccinia virus mouse tail lesion model [7,8]. HPMPC (cidofovir, CDV) was shown to be active in lethal aerosol or intranasal cowpox infections [9,10] when given by parenteral injection. CDV was also active when given as an aerosol in cowpox-infected mice [11]. However, CDV given intravenously has substantial nephrotoxicity which limits its dose to 5 mg/kg/week. In a smallpox outbreak, it would not be possible to use an intravenous drug with significant nephrotoxicity widely. An oral drug with less toxicity which could be delivered from a stockpile and self administered would be much more desirable for emergency use.

## 3. Enhancing the Oral Activity of Antiviral Nucleosides: Early Development of the Alkoxyglycerol Esterification Approach

Our early approach to antiviral drug design was to attempt to enhance cellular drug uptake by disguising antiviral nucleosides as diacylphosphatidylglycerol nucleoside analogs. When liposomal preparations containing phosphatidyl nucleoside conjugates such as 1,2-dipalmitoylphosphatidyl-2′,3′-dideoxyguanosine (DPP-ddG) were prepared and evaluated by intraperitoneal administration to woodchucks infected with woodchuck hepatitis virus, a significant antiviral effect was observed while an equimolar dose of ddG itself had little effect [12]. While this study clearly demonstrated the utility of a liposome phosphatidyl-linked antiviral nucleoside to target a liver infection, parenteral administration was required and subsequent experiments revealed that DPP-ddG, phosphatidyl-AZT and phosphatidyl analogs of other nucleosides were poorly absorbed after oral administration in mice. Early nucleoside analog antiviral agents like acyclovir (ACV) and ganciclovir (GCV) exhibited low oral bioavailability and we were interested in making analogs which were more active orally. Phospholipids are a part of normal dietary intake and specialized absorption pathways for lipids exist [13]. Humans ingest roughly 2 to 8 grams of phospholipid daily, which represents 1 to 10% of total daily fat intake, and phosphatidylcholine (lecithin) is the most common phospholipid in the diet. We took the approach to orally active lipid antivirals by basing the drug design on lysophosphatidylcholine (LPC), a partially degraded phospholipid formed in the small intestinal lumen from phosphatidyl-choline by the action of pancreatic phospholipase A_2._ Realizing that a substantial percentage of LPC is known to be absorbed intact [14,15], we replaced the acyl ester bond at the *sn*-1 glycerol position of LPC with an ether linkage to block hydrolysis of the acyl group by pancreatic lysophospholipase during absorption. Another advantage is that the alkyl ether analog is much more stable on storage than LPC which contains an acyl ester. Finally, the *sn*-2 hydroxyl of glycerol in LPC was replaced with a hydrogen atom in order to prevent reacylation by lysophosphatidylcholine acyltransferases which would result in the formation of diacylphosphatidyl nucleosides which we knew were poorly absorbed. We applied the design strategy to several nucleoside phosphate analogs using the hexadecyloxypropyl ester since this is the most common acyl substituent of LPC.

### 3.1. Oral Activity of Acyclovir (ACV) and HDP-P-ACV in Woodchuck Hepatitis B and HSV-1 Infections in vivo

**Figure 1:**
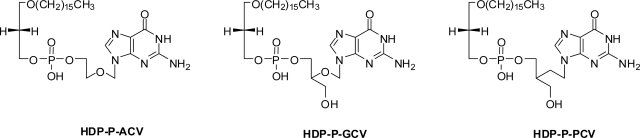
Structure of HDP-P esters of Acyclovir, Ganciclovir and Penciclovir.

ACV-triphosphate is an effective inhibitor of hepatitis B virus (HBV) polymerase but ACV itself is not highly active in patients infected with HBV, indicating that ACV is not adequately phosphorylated in the liver to its triphosphate and does not show any anti-HBV activity [16,17]. We synthesized HDP-P-ACV (Figure 1) and found that the lipid prodrug has significantly enhanced activity against hepatitis B virus replication in 2.2.15 cells compared to ACV. Metabolic studies showed that the increased activity was due in part to much higher intracellular levels of ACV mono-, di-, and triphosphates *versus* those levels found with exposure to acyclovir [18]. Using radiolabeled HDP-P-ACV, we found that the HDP-P-ACV was orally bioavailable and gave rise to excellent liver exposure in mice. Analysis of plasma showed that about half of the radioactive drug was circulating as HDP-P-ACV, the intact prodrug [18]. Oral HDP-P-ACV also proved to be effective *in vivo* against woodchuck hepatitis virus, providing a 2 log reduction in viral load while ACV at the maximal tolerated dose had no significant effect [19]. We also treated mice infected with HSV-1 with oral ACV or HDP-P-ACV. On a molar basis HDP-P-ACV, the lipid prodrug, was 2.4 times more active orally than ACV in preventing death from acute HSV-1 infection in mice [20].

### 3.2. Oral Activity of HDP-P-GCV and HDP-P-PCV in HSV-1 Encephalitis

Encouraged by these results with ACV, we synthesized and tested the HDP-phosphate esters of penciclovir (PCV) and ganciclovir (GCV), two agents known to have poor oral bioavailability (Figure 1). HDP-P-GCV was orally active against murine CMV and HSV-1 infections in mice, with antiviral activity equivalent to or greater than oral GCV [21]. With PCV, *in vitro* data showed that HDP-P-PCV was somewhat less potent than PCV against HSV-1. However, unmodified PCV showed little effect on mortality from intranasal HSV-1 infection in mice when given orally. In contrast, oral dosing of HDP-P-PCV (120 mg/kg, twice daily) showed a significant effect, reducing mortality in the infected mice from 87 to 40% [21].

## 4. Synthesis of HDP-CDV

We next turned out attention to CDV, HPMPA, and the acyclic nucleoside phosphonates (ANPs). The ANPs can be regarded as close analogs of nucleoside monophosphates, except that they possess a metabolically stable phosphorus-carbon bond and lack a complete ribose or deoxyribose functionality. Unlike conventional nucleosides, ANPs do not require the often rate-limiting initial phosphorylation step for activation. As a result, ANPs like CDV and HPMPA are fully active against thymidine kinase or UL-97 mutant viruses which are resistant to acyclovir or ganciclovir. After conversion of ANPs to the active metabolite inside cells (ANP-diphosphate), the metabolites are generally retained inside cells for prolonged periods of time, which leads to more convenient dosing regimens [22]. However, ANPs have poor cellular permeation and oral bioavailability due to the charges on the phosphonic acid at physiologic pH levels. Another drawback involves their tendency to concentrate in the kidney proximal tubule, resulting in nephrotoxicity [22]. The next section describes the application of the alkoxyalkyl ester strategy to ANPs.

### Synthesis of HDP-Cyclic-CDV and HDP-CDV

**Figure 2:**
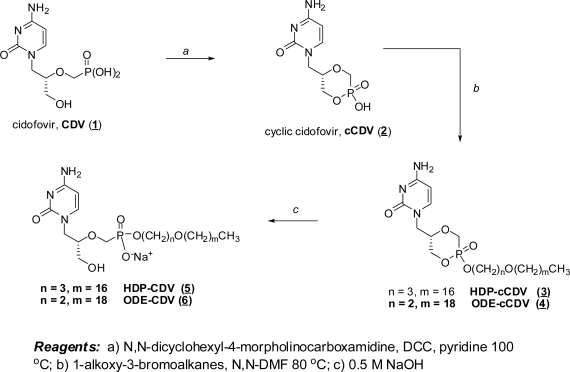
Synthesis of HDP-CDV and ODE-CDV.

Building on early successes with HDP-P esters of acyclovir and ganciclovir, we synthesized the hexadecyloxypropyl and octadecyloxyethyl esters of CDV and cyclic CDV as shown in Figure 2 [23]. Briefly, CDV (**1**) was cyclized with dicyclohexylcarbodiimide to cyclic CDV (**2**) and esterified with 3-hexadecyloxy-1-propanol or 2-octadexyloxy-1-ethanol by the Mitsunobu reaction [24], yielding HDP-cyclic CDV (**3**) or ODE-cyclic CDV (**4**). The cyclic diesters were exposed to 0.5 N NaOH to open the ring, yielding HDP-CDV (**5**) or ODE-CDV (**6**). Alternative methods of synthesis of the alkoxyalkyl nucleoside phosphonates which are more suitable for scale up have been reported by Beadle and coworkers [25].

## 5. *In vitro* Antiviral Activity of HDP-CDV and ODE-CDV against Variola, Monkeypox, Cowpox and Vaccinia Viruses

Table 1 shows the results of antiviral studies done *in vitro* with variola Bangladesh, monkeypox Zaire and cowpox Brighton in Vero 76 cells or MK2 cells *in vitro* [26]. The EC_50_ values of HDP-CDV against variola ranged from 0.04 to 0.1 μM; octadecyloxyethyl-CDV (ODE-CDV) was somewhat more active at 0.01 to 0.03 μM. The alkoxyalkyl esters were 25- to 910-fold more active than CDV. Similar results were noted with monkeypox and cowpox viruses *in vitro* (Table 1). HDP-CDV and ODE-CDV were tested *in vitro* against vaccinia virus. The EC_50_ for CDV was 46.2 μM compared with 0.8 μM for HDP-CDV, an increase in antiviral activity of 58-fold. ODE-CDV was more active with an EC_50_ of 0.2 μM, a 231 fold increase [27]. These results encouraged further evaluation of HDP-CDV and ODE-CDV as possible treatments for disease caused by vaccinia or variola infections.

## 6. Mechanism of Action

### 6.1. Mechanism of Increased Antiviral Activity of HDP-CDV

**Figure 3:**
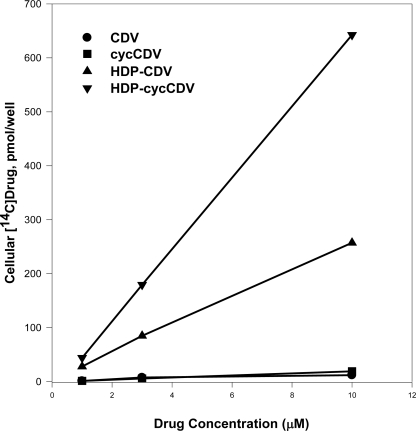
Uptake of ^14^C-labeled CDV, cyclic-CDV, HDP-cyclic-CDV and HDP-CDV in MRC-5 Human Lung Fibroblasts. Adapted from Reference 31.

The increase in antiviral activity noted with HDP-CDV compared to CDV itself was unusually large (100-fold or more). To investigate the reason for this, we next evaluated the cellular uptake and metabolism of [2-^14^C]-CDV and HDP-[2-^14^C]-CDV in MRC-5 human lung fibroblasts, a common host cell for poxvirus study [28]. At four hours, the cellular radioactive drug content observed after exposure to 1 μM CDV was only 1.2 *versus* 28 picomoles/well with 1.0 μM HDP-CDV. At concentrations of 3 and 10 μM, drug uptake of HDP-CDV was 77 and 245 picomoles/well, an increase of 11- to 23-fold *versus* CDV (Figure 3). At 3 and 10 μM, the uptake of HDP-cCDV was approximately twice that of HDP-CDV and 32-fold greater than observed with cCDV (Figure 3). The mechanism of uptake has not been studied extensively, but it is believed that the HDP-CDV internalized after insertion into the plasma membrane bilayer outer leaflet as is typical for lysophospholipids. Transfer to the internal leaflet is probably spontaneous but may also be catalyzed by flippases. This process has not been studied in detail. In the cells phospholipase C or other phosphatases as yet unidentified, release CDV which is converted to the active metabolite by cellular enzymes. Conversion of HDP-CDV to CDV diphosphate (CDVpp), an analog of deoxycytidine triphosphate, was measured by HPLC. Cells exposed to CDV and HDP-CDV for 48 hours had 1.8 and 184 picomoles of CDVpp, respectively; a difference of 100-fold [28]. Thus, it seems clear that increased antiviral activity of HDP-CDV is due to increased cellular uptake and conversion to CDVpp, the inhibitor of poxvirus DNA polymerase.

### 6.2. Mechanism of Inhibition of Vaccinia DNA Polymerase by CDV Diphosphate

The mechanism of inhibition of the vaccinia polymerase had not been studied previously. Using the purified vaccinia E9L DNA polymerase and different primer template pairs, Wendy Magee and David Evans found that CDV was incorporated into the growing DNA strand opposite template G’s but at a lower catalytic efficiency than dCTP [29]. CDV-terminated primers could be extended at the next addition step, but these CDV+1 reaction products are not good substrates for further synthesis. While CDV can be excised from the primer 3′ terminus by exonuclease of vaccinia virus polymerase, DNAs which have CDV as the penultimate 3′ residue were found to be completely resistant to exonuclease cleavage [29]. In addition, and perhaps more importantly, when CDV is present in the template strand, the vaccinia DNA polymerase is not able to bridge the CDV base, resulting in chain termination which prevents further rounds of replication; similar results were noted when HPMPA was incorporated into the template strand [30]. Interestingly, HPMPA, the adenine analog of HPMPC, inhibits exclusively by the latter mechanism. Thus, there are several important mechanisms by which CDV and HPMPA inhibit vaccinia virus replication.

## 7. Early Studies of the Pharmacology and Toxicology of HDP-CDV in Mice

Equimolar doses of ^14^C-labeled CDV (5.6 mg/kg intraperitoneal) and HDP-CDV (10 mg/kg oral or intraperitoneal) were administered to mice [31]. After oral administration, HDP-CDV and metabolites in plasma reached their highest level, 2.34 μM, at three hours while intraperitoneal CDV showed a Cmax of 0.84 μM at one hour. Comparison of the oral and parenteral areas under curve (AUC) indicated that the relative oral bioavailability of HDP-CDV was 88% [31]. The oral bioavailability of CDV in rats has been reported to be less than 3%. HDP-CDV persists in plasma following absorption in mice with a T_½_ of 14.9 hours and is converted to CDV and metabolites in the tissues [31]. HDP-CDV is stable in plasma for several days at 37 °C and the plasma CDV noted after administration of HDP-CDV is a result of tissue metabolism.

The tissue distribution of CDV, HDP-CDV and metabolites was determined using ^14^C-labeled drugs and the Cmax and area under curve (AUC_0–72hr_) of drug and metabolites was calculated (Table 2). Several notable trends were apparent. The Cmax for intraperitoneal CDV in kidney was 180 nanomoles/gm, 30-times that observed for oral HDP-CDV. When CDV is given intraperitoneally, most of the radioactive CDV appeared in the kidney and very little CDV was noted in brain, heart, liver, lung and spleen. In contrast, oral HDP-CDV delivered large amounts of the drug and metabolites to liver and and greater exposures in other tissues including brain. In kidney, HDP-CDV AUC was only 19% of that observed with parenteral CDV, suggesting that the high kidney C_max_ and AUC noted with parenteral CDV is responsible for its nephrotoxicity [31].

Early oral toxicity studies with HDP-CDV showed that single doses of 100 mg/kg could be used safely [32]. However, significant weight loss and some mortality were noted at doses of 30 mg/kg/day for 7 to 14 days which was apparently due to gastrointestinal toxicity. No kidney or liver toxic effects were apparent (Hostetler, unpublished observations, 2002). We were able to use 1 to 10 mg/kg/day doses of HDP-CDV for up to 21 days in various animal models of viral disease.

## 8. Antiviral Activity in Poxvirus Disease

### 8.1. Studies in Lethal Animal Models of Orthopoxvirus Disease

In 2002, HDP-CDV was first tested in lethal cowpox virus infection in mice [33]. Full protection was noted when HDP-CDV was given orally at 5 mg/kg for five days and treatment was started two days post infection with 5 × 10^7^ pfu of intranasal cowpox virus. Oral CDV was inactive, but a 100 mg/kg dose of intraperitoneal CDV provided full protection [33].

In 2004, Mark Buller and coworkers [34] reported that HDP-CDV and ODE-CDV were active *in vitro* against ectromelia. When given orally to mice infected with ectromelia virus by small particle aerosol at 2.3 × 10^4^ pfu/mouse, HDP-CDV and ODE-CDV were near fully protective by oral doses of 5 mg/kg and 10 mg/kg, respectively, when given four hours after infection and continued daily for five days [34]. Both HDP-CDV and ODE-CDV were effective in reducing mortality at 10 to 12.5 mg/kg for five days when given orally prior to or one, two or three days following intranasal infection with CPX or VV in BALB/c mice [35]. Mice treated 24 h after infection with the IHD strain of vaccinia virus with a single dose of 100, 50 or 25 mg/kg of HDP-CDV showed highly significant protection with zero to 20% mortality *versus* 100% mortality seen in untreated, infected mice [32]. HDP-CDV given orally also provides protection from infection with rabbitpox [36].

In 2008, Parker and coworkers [37] studied various infectious doses of ectromelia virus and found that the dose of HDP-CDV required for full protection from mortality varies with the dose of infectious virus. At high viral input doses (500–5,000 pfu/mouse), 8 mg/kg HDP-CDV for five days provided complete protection. With 50 pfu infectious doses of ectromelia virus, 2 mg/kg of HDP-CDV for five days provided 87% protection while low dose infections of less than 5 pfu of input virus required only 2 mg/kg of HDP-CDV for 5 days to achieve 100% protection [37].

### 8.2. Results in Human Vaccinia Infection

Recently a patient who was vaccinated for smallpox and later found to have leukemia developed a disseminated vaccinia virus infection. After being treated for 21 days with vaccinia immune globulin, ST246 and imiquimod with little beneficial effect, the patient cleared the vaccinia infection rapidly after the addition of oral HDP-CDV (CMX001) to the treatment regimen [38]. Although this patient was cured by treatment with multiple agents, it seems quite clear from the clinical description that improvement coincided with the addition of oral therapy with CMX001.

## 9. Current Status of Development of HDP-CDV (CMX001)

CMX001 is under consideration as an agent for inclusion in the Strategic National Stockpile for emergency use in the event of a release of variola or for complications of smallpox vaccination. In addition to its antiviral activity against the orthopoxviruses, HDP-CDV has been shown to be active as an antiviral against all double stranded DNA (dsDNA) virus infections including human CMV [39]; HSV-1, HSV-2 and EBV [40], various strains of adenovirus [41], BK virus [42], orf [43], JC virus and human papilloma virus [44]. Currently, CMX001 has completed Phase I clinical trials in normal volunteers and Phase II trials are ongoing in CMV infections in stem cell transplant patients (NCT00942305) and BK virus infection in kidney transplant patients (NCT00793598). In addition, CMX001 has been used successfully in a number of emergency IND (E-IND) applications in patients having infections with CMV, HSV, adenovirus, BK virus and JC virus infections sponsored by Chimerix Inc., the licensee. Finally, an expanded access trial entitled “A Multicenter, Open-label Study of CMX001 Treatment of Serious Diseases or Conditions Caused by dsDNA Viruses” is also open (NCT01143181).

## 10. Conclusions

HDP-CDV is a novel ether lipid antiviral which is orally active and lacks the nephrotoxicity of CDV itself. Increased oral bioavailability and increased cellular uptake, facilitated by the lipid portion of the molecule, is responsible for the improved activity profile. It is a highly effective agent against a variety of orthopoxvirus infections in animals including vaccinia, cowpox, rabbitpox and ectromelia. Its activity was recently demonstrated in disseminated vaccinia infection in man after HDP-CDV (CMX001) was added to a multiple drug regimen. In addition to the activity against orthopoxviruses, HDP-CDV (CMX001) is active against all dsDNA viruses including CMV, HSV-1, HSV-2, EBV, adenovirus, BK virus, orf, JC, and human papilloma viruses, and is in clinical development as a treatment for human infections with these viruses.

## Figures and Tables

**Table 1:** Antiviral Activity of HDP-CDV and ODE-CDV Against Variola, Monkeypox and Cowpox, *in vitro*.

	**Variola Bangladesh**		**Monkeypox Zaire**		**Cowpox Brighton**	
**Drug**	**V76**	**MK2**	**V76**	**MK2**	**V76**	**MK2**
**CDV**	27.3	10.2	4.6	4.3	36.5	7.0
**HDP-CDV**	0.10	0.04	0.07	0.13	0.10	0.008
**ODE-CDV**	0.03	0.01	0.006	0.006	0.04	0.009

Data are μM EC_50_ values in Vero 76 cells (V76) or MK2 cells infected with Variola Bangladesh, Monkeypox Zaire or Cowpox Brighton. Adapted from Reference [26].

**Table 2:** Comparison of areas under curve with oral HDP-[2-^14^C]-CDV and intraperitoneal [2-^14^C]-CDV in mouse tissues.

	**AUC_0-72hr_**		**AUC Ratio**
**Tissue**	**CDV**	**HDP-CDV**	**H/C**
Brain	0.87	3.96	4.6
Heart	3.46	14.26	4.1
Liver	39.6	1366.0	34.5
Lung	6.27	49.9	8.0
Kidney	1093.0	203	0.19
Spleen	9.92	53.2	5.4

Data are nmol/gm.hrs after a single oral dose of 10 mg/kg HDP-[2-^14^C]-CDV or a molar equivalent intraperitoneal dose of [2-^14^C]-CDV (5.6 mg/kg). Ratio column abbreviation: H = HDP-CDV, C = CDV. Adapted from Reference [31].
